# Suicidal tendencies in university students during the COVID-19 outbreak

**DOI:** 10.1192/j.eurpsy.2021.1554

**Published:** 2021-08-13

**Authors:** E. Nikolaev

**Affiliations:** Social And Clinical Psychology, Ulianov Chuvash State University, Chebokasry, Russian Federation

**Keywords:** suicidal ideations, University Students, COVID-19 outbreak

## Abstract

**Introduction:**

Suicidogenic effects COVID-19 pandemic are expected to reveal themselves not immediately, but within a longer period.

**Objectives:**

To evaluate the prevalence of suicidal tendencies in university students during the COVID-19 outbreak and specify the psychosocial characteristics of the students with a low anti-suicidal barrier to mitigate their suicide risks.

**Methods:**

The research was done via an on-line survey, which covered 536 students of both sexes (aged 21.46±2.95), who studied in Russian universities and who filled in a structured questionnaire during their distance learning due to COVID-19 outbreak.

**Results:**

We revealed that 11.38% of the respondents (57.47% of whom are males) with a low anti-suicidal barrier showed suicidal tendencies by allowing the possibility of committing a suicide in a certain situation. Among them were more Russian students than international ones (p=.0272). They also certainly exceeded the students with the developed anti-suicidal barrier in taking alcohol (p=.0126), in underestimating their own health (p=.0053), in expressing happiness (p=.0001), and in degree of religious belief (p=.0001). They perceived the situation associated with the COVID-19 outbreak with a more strongly manifested anxiety due to the fear of their own infection with coronavirus (p=0.0347). At the same time, they acted less responsibly in following personal restrictive measures aimed to reduce the risk of infection (p=.0002).

**Conclusions:**

Students with suicidal tendencies during the COVID-19 outbreak present a risk group in COVID-19 spread and infection. The pandemic can intensify anti-vital sufferings and enhance the risk of committing suicide in individuals with suicidal tendencies, which should be taken into account in prevention programs.

